# Itch as Major Mediator of Effect of Tofacitinib on Health-Related Quality of Life in Psoriatic Arthritis: A Mediation Analysis

**DOI:** 10.3390/jcm10184081

**Published:** 2021-09-09

**Authors:** Peter C. Taylor, Andrew G. Bushmakin, Joseph C. Cappelleri, Pamela Young, Rebecca Germino, Joseph F. Merola, Gil Yosipovitch

**Affiliations:** 1 Rheumatology and Musculoskeletal Sciences, Nuffield Department of Orthopaedics, University of Oxford, Oxford OX3 7LD, UK; 2 Pfizer Inc, Groton, CT 06340, USA; andrew.g.bushmakin@pfizer.com (A.G.B.); joseph.c.cappelleri@pfizer.com (J.C.C.); 3 Pfizer Inc, Collegeville, PA 19426, USA; pamela.young2@pfizer.com; 4 Pfizer Inc, New York, NY 10017, USA; rebecca.germino@pfizer.com; 5 Brigham and Women’s Hospital, Harvard Medical School, Boston, MA 02115, USA; jfmerola@bwh.harvard.edu; 6 Miller School of Medicine, University of Miami, Miami, FL 33146, USA; yosipog@gmail.com

**Keywords:** itch, psoriatic arthritis, quality of life, mediation modeling

## Abstract

Patients with psoriatic arthritis (PsA) experience impaired health-related quality of life (HRQoL). Tofacitinib is an oral Janus kinase inhibitor for the treatment of PsA, which has been associated with improvements in dermatologic endpoints in patients with PsA. To assess the extent to which tofacitinib affects patient HRQoL via improvements in dermatologic symptoms, including itch, data were pooled from patients with PsA who received tofacitinib in phase III studies (NCT01866668 and NCT01882439). Mediation modeling assessed the indirect effects (via Itch Severity Item [ISI] and Physician’s Global Assessment of Psoriasis [PGA-PsO]) and direct effects (via all other factors) of tofacitinib treatment on dermatology-specific HRQoL (measured by Dermatology Life Quality Index [DLQI]). In the initial model, the treatment effect on DLQI was largely mediated by itch (ISI; *p* < 0.0001) and PGA-PsO (*p* < 0.01). The model was re-specified to assess the indirect effects only of itch and PGA-PsO on DLQI. Here, 17.7% of the treatment effect on DLQI was attributable to PGA-PsO (*p* = 0.0006), and 82.3% to itch (*p* < 0.0001). Tofacitinib-dependent improvements in DLQI were primarily mediated by itch relief, in addition to improvements in PGA-PsO.

## 1. Introduction

Psoriatic arthritis (PsA) is a chronic, systemic inflammatory disease with signs and symptoms across multiple domains, including enthesitis and dactylitis, axial disease, cutaneous manifestations, and pain, swelling, and stiffness in the joints [1,2,3,4]. The global prevalence of PsA is approximately 0.01–0.19% in the general population [5], but develops in up to 30% of individuals with psoriasis [6]. It has been shown to substantially impact the physical and mental state of patients [7], and leads to impaired health-related quality of life (HRQoL) [8].

Dermatologic symptoms have been shown to have a substantial impact on patient quality of life [9]. A retrospective study of 637 patients with PsA found that >80% of patients were reported to possess joint and active skin involvement in PsA, which was associated with a more severe clinical profile and poorer outcomes compared with those who did not have skin symptoms [10]. It was also previously shown that clinical improvements in skin symptoms following treatment were associated with improvements in physical and mental health in patients with PsA [11]. In a qualitative analysis, 474 patients with PsA completed PsA Impact of Disease (PsAID) questionnaires (PsAID-9 and PsAID-12) to assess the impact of PsA on their lives, ranking ‘skin problems’ as the third most important domain of health [12].

Itch has been highlighted as a key dermatologic symptom of PsA; in a multinational, population-based survey of 3426 patients with psoriasis and/or PsA, itch was the most commonly reported symptom related to skin disease [4]. Moreover, 40% of patients with PsA rated itch as the most bothersome skin complaint [4]. The ability to reduce dermatologic symptoms, such as itch, and improve patient HRQoL is a key focus in the development of treatments for PsA.

Janus kinase (JAK)-1 has been shown to be a driver of chronic itch; therefore, JAK inhibition represents a potential antipruritic strategy [13]. There has been increasing interest in the involvement of the JAK/signal transducer and activator of transcription (STAT) pathway in pronociceptive signaling, focusing on the ability of JAK inhibitors to modulate pain [14]. Given that itch is also considered to be a form of nociception [15], these findings provide further support of the potential for JAK inhibitors to relieve itch.

Tofacitinib is an oral JAK inhibitor for the treatment of PsA. The efficacy and safety of tofacitinib 5 and 10 mg twice daily (BID) have been previously demonstrated in two phase III randomized controlled trials in patients with active PsA and an inadequate response to either conventional synthetic disease-modifying antirheumatic drugs (csDMARDs) or tumor necrosis factor inhibitors (TNFi) (OPAL Broaden and OPAL Beyond; NCT01877668 and NCT01182439, respectively). In both studies, 47–61% of patients treated with tofacitinib met the primary endpoint of ACR20 at Month 3, compared with placebo (24–33%) [16,17]. Similarly, in patients treated with tofacitinib, mean changes from baseline in HAQ-DI score at Month 3 were significantly greater, compared with placebo [16,17]. Patients receiving tofacitinib experienced greater improvements in various dermatologic endpoints, including Patient Global Assessment of Disease Activity and Patient’s Global Joint and Skin Assessment at Month 3, compared with placebo [18,19]. A post-hoc analysis of data from primary studies suggested that in patients with active PsA who had an inadequate response to conventional synthetic disease-modifying antirheumatic drugs (csDMARDs) or tumor necrosis factor inhibitors (TNFi), tofacitinib 5 or 10 mg BID improved dermatologic patient-reported outcomes and dermatology-related quality of life at Month 3, compared with placebo [20]. Specifically, patients demonstrated greater improvements (*p* ≤ 0.05) in itch (as assessed by Itch Severity Item [ISI]) and dermatology-specific HRQoL (as assessed by Dermatology Life Quality Index [DLQI]) at Month 3 with tofacitinib versus placebo [20]. Furthermore, one of the questions in the DLQI assessment focuses on patients’ scoring of itch and pain related to skin symptoms [21], and improvements from baseline to Month 3 for this particular score were observed with both doses of tofacitinib versus placebo [20].

Mediation modeling is a statistical methodology used to identify the mechanisms underlying inter-relationships between an independent variable (e.g., a treatment) and a dependent variable (e.g., an outcome, such as HRQoL), via the inclusion of a third explanatory (mediator) variable [22]. Mediation analysis has previously been used to study the mechanisms by which tofacitinib affects itch in patients with psoriasis [23], as well as overall patient treatment satisfaction in patients with ulcerative colitis [24].

Here, we used mediation modeling to investigate the extent to which clinical improvements in skin symptoms, including itch, mediate improvements in dermatology-specific HRQoL (as measured by DLQI) in patients with active PsA treated with tofacitinib.

## 2. Materials and Methods

This analysis included data collected from two randomized, placebo-controlled, double-blind phase III studies of patients with active PsA. OPAL Broaden (NCT01877668) had a duration of 12 months, and enrolled patients who were TNFi-naïve and had a previous inadequate response to ≥1 csDMARD [16]. Patients were randomized to receive tofacitinib 5 mg BID, tofacitinib 10 mg BID, adalimumab 40 mg subcutaneous once every 2 weeks, or placebo. Patients who received placebo advanced to tofacitinib 5 or 10 mg BID at Month 3. OPAL Beyond (NCT01882439) had a duration of 6 months, and enrolled patients who had a previous inadequate response to ≥1 TNFi [17]. Patients were randomized to receive tofacitinib 5 mg BID, tofacitinib 10 mg BID, or placebo. Patients who received placebo advanced to tofacitinib 5 or 10 mg BID at Month 3. In both studies, all patients were treated continuously with a stable dose of a single csDMARD. In the current analysis, only data for patients treated with tofacitinib 5 mg BID or placebo up to Month 3 were included.

Mediation modeling was used to identify mechanisms underlying an observed relationship between an independent variable (e.g., tofacitinib treatment) and an outcome (e.g., improvements in DLQI) via the inclusion of a third explanatory variable (e.g., itch). The mediation model included data for the following assessments: DLQI, ISI, and PGA-PsO. DLQI is a general dermatology questionnaire consisting of 10 items to assess patient-reported HRQoL [21]; DLQI scores range from 0 to 30, where scores of 0–1 are defined as “no effect on patient’s life” and scores of 21–30 are defined as “extremely large effect on patient’s life” [25]. ISI is a single-item numeric rating scale, which allows patients to rate the severity of their itching due to psoriasis in the previous 24 h [26]. The ISI scale ranges from 0 (“no itching”) to 10 (“worst possible itching”). PGA-PsO is a 5-point scale that takes into account erythema, induration, and scaling across all psoriatic lesions [27]. Average scores for erythema, induration, and scaling are determined separately, each with their own 5-point scale. The average value of all three severity scores is calculated and rounded to the nearest whole number, with overall PGA-PsO scores ranging from 0 (“clear”) to 4 (“severe”).

**Figure 1:**
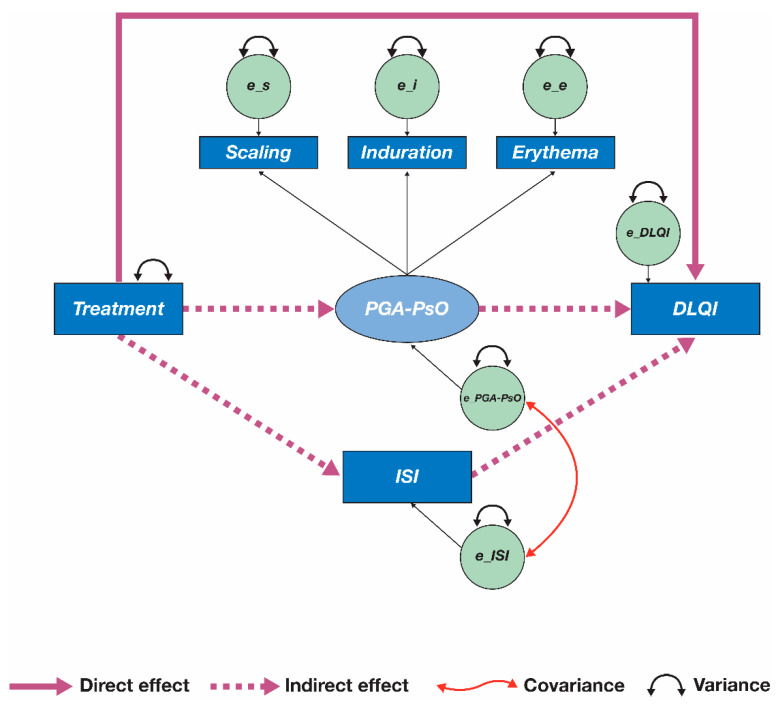
Initial mediation model. Treatment is represented by a binary variable (tofacitinib 5 mg twice daily [value of 1] versus placebo [value of 0]); the error terms for Physician Global Assessment of Psoriasis (e_PGA-PsO) and Itch Severity Item (e_ISI) are connected by a curved two-headed arrow, indicating that these two variables are allowed to covary (meaning that the variables ISI and PGA-PsO are not assumed to be independent). A curved two-headed arrow pointing to the same variable represents variance. DLQI, Dermatology Life Quality Index; e_DLQI, error term for DLQI; e_e, error term for erythema; e_i, error term for induration; e_s, error term for scaling.

An overview of the initial mediation model is shown in Figure 1. For each assessment (DLQI, ISI, and PGA-PsO [represented by erythema, induration, and scaling]), a mean value across Months 1 and 3 was calculated for every patient with available data. The main analyses included data pooled from both OPAL Broaden and OPAL Beyond. Additional analyses were also independently performed on data from each study. In the initial mediation model, treatment was defined as the independent (explanatory) binary variable, with tofacitinib 5 mg BID assigned a value of 1 and placebo assigned a value of 0 (Figure 1). DLQI was defined as the dependent (outcome) variable, and ISI and PGA-PsO were defined as mediators (Figure 1). PGA-PsO was a latent variable represented by mean scores for erythema, induration, and scaling. A previous analysis of data from a phase IIb study of patients with psoriasis demonstrated that tofacitinib mostly affects itch directly, with a smaller proportion of the effect mediated by improvements in erythema, induration, and scaling (PGA-PsO) [23]. As such, itch and PGA-PsO were treated as distinct mediators in the current analysis, but were not assumed to be independent.

In the initial model, the effects of tofacitinib treatment on DLQI, mediated by ISI and PGA-PsO, were designated as indirect effects. All other effects of tofacitinib treatment on DLQI not attributable to these two mediators were designated as direct effects. The mediation model could be re-specified based on the results of the initial mediation model. Effects with *p* < 0.05 were considered statistically significant.

## 3. Results

### 3.1. Patients

Overall, data for 468 patients were available for the analyses (210 patients from OPAL Broaden and 258 patients from OPAL Beyond). Baseline demographics and disease characteristics for all patients included in both primary studies have been previously published [16,17,20].

### 3.2. Initial Mediation Model

**Figure 2:**
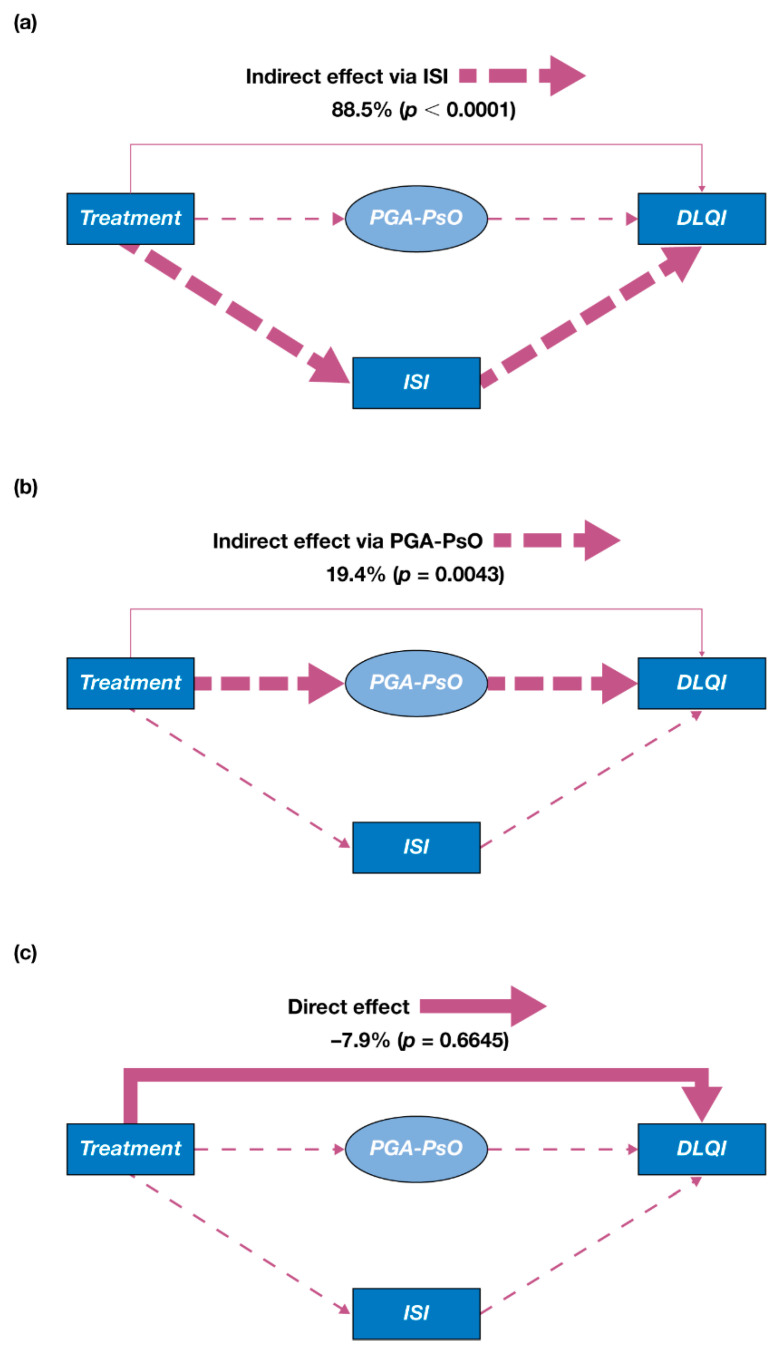
Effects of tofacitinib treatment on DLQI in the initial mediation model (pooled data from OPAL Broaden and OPAL Beyond). (**a**) Indirect effect via itch (measured by ISI); (**b**) indirect effect via PGA-PsO; (**c**) direct effect. Treatment was represented by a binary variable (tofacitinib 5 mg twice daily [value of 1] versus placebo [value of 0]); the model included mean scores of each outcome from Months 1 and 3 for every patient with available data from OPAL Broaden or OPAL Beyond; PGA-PsO was a latent variable represented by means of erythema, induration, and scaling.

In the analyses of data pooled from both OPAL Broaden and OPAL Beyond, the effect of tofacitinib treatment on DLQI was largely mediated by itch (measured by ISI) and PGA-PsO (Figure 2). The majority of this indirect effect was attributable to improvements in itch (88.5%, *p* < 0.0001; Figure 2a); however, the indirect effect mediated via improvements in PGA-PsO was also statistically significant (19.4%, *p* = 0.0043; Figure 2b). The effect of tofacitinib treatment on DLQI due to factors other than itch and PGA-PsO (i.e., the direct effect) was small and not statistically significant (−7.9%, *p* = 0.66; Figure 2c). Independent analyses of data from OPAL Broaden (Figure S1) and OPAL Beyond (Figure S2) produced consistent results.

### 3.3. Re-Specified Mediation Model

As the direct effect observed in the initial model was small and not statistically significant, the model was re-specified to exclude the direct effect of tofacitinib treatment on DLQI. Therefore, only the indirect effects mediated via ISI and PGA-PsO were assessed; the independent and dependent variables remained the same as in the initial model.

**Figure 3:**
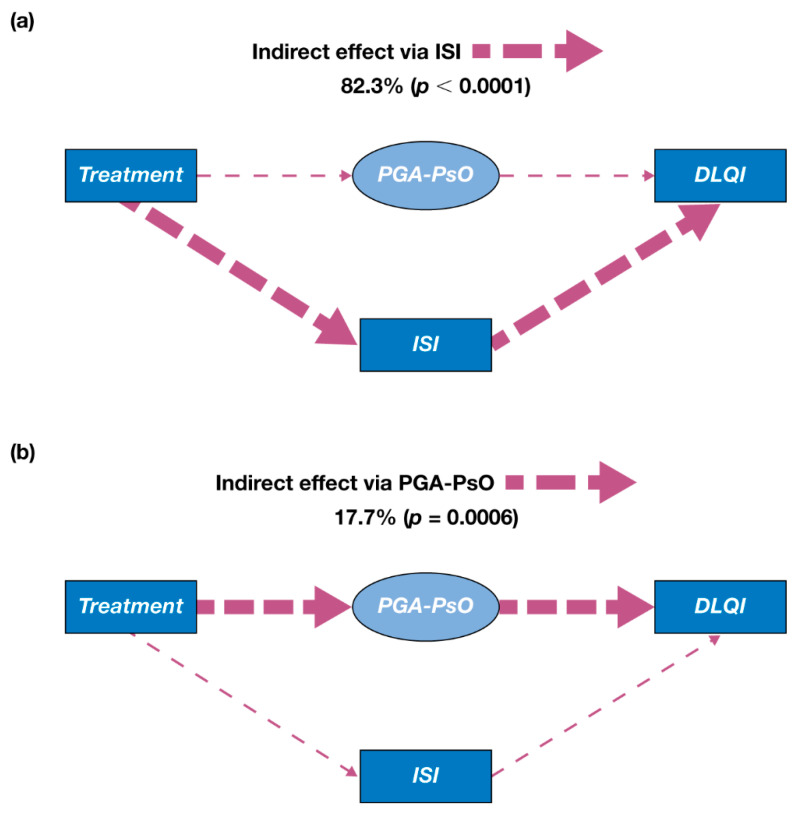
Effects of tofacitinib treatment on DLQI in the re-specified mediation model (pooled data from OPAL Broaden and OPAL Beyond). (**a**) Indirect effect via itch (measured by ISI); (**b**) indirect effect via PGA-PsO. Treatment was represented by a binary variable (tofacitinib 5 mg twice daily [value of 1] versus placebo [value of 0]); the model included mean scores of each outcome from Months 1 and 3 for every patient with available data from OPAL Broaden or OPAL Beyond; PGA-PsO was a latent variable represented by means of erythema, induration, and scaling.

When data pooled from both OPAL Broaden and OPAL Beyond were analyzed using the re-specified model, results were similar to the initial mediation model. Overall, 82.3% (*p* < 0.0001) of the effect of tofacitinib treatment on DLQI was attributable to improvements in itch, as measured by ISI (Figure 3a), and 17.7% (*p* = 0.0006) was attributable to improvements in PGA-PsO (Figure 3b). Similar results were observed when data from OPAL Broaden (Figure S3) and OPAL Beyond (Figure S4) were analyzed independently.

## 4. Discussion

This analysis aimed to identify and quantify the roles of itch and PGA-PsO on the effect of tofacitinib treatment on dermatology-specific HRQoL (as measured by DLQI) in patients with PsA. Using mediation modeling, we found that itch and PGA-PsO accounted for the majority of the overall effect of tofacitinib treatment on DLQI scores, with itch being the main contributor. This result was observed when data were pooled from TNFi-naïve patients (OPAL Broaden) and patients who had an inadequate response to TNFi (OPAL Beyond), and findings were consistent when data from each study were analyzed independently.

There is a drive towards improving the shared decision-making process between patients with PsA and their physicians; patients’ quality of life and the impact of treatment is a key focus [28]. Therefore, it is important to understand what aspects of the disease affect quality of life, and the mechanism(s) by which treatment can improve this. It is recognized that PsA has a substantial effect on quality of life, with negative impacts including impairment of physical and social functioning, mental state, and sleep quality [7]. Dermatologic symptoms, such as itch, are commonly reported by patients with PsA, and these have been shown to have an impact on patients’ lives [9,12,28].

A survey of patients who experienced chronic itch or chronic pain for >6 weeks, not specific to a certain disease type, found that itch had a major influence on HRQoL, comparable to the impact of pain [29]. In a survey of patients with psoriasis and/or PsA, 43% and 40% of patients with psoriasis and PsA, respectively, rated itch as the most bothersome skin symptom [4]. Furthermore, itch was reported as the most important factor contributing to disease severity in psoriasis (38% of patients), and the second most important factor in PsA (18% of patients) [4]. Further research is required to assess the specific impact that itch has on patients with PsA; however, various studies have investigated this in patients with psoriasis. Using a questionnaire designed to evaluate the clinical characteristics of itch, 24% of patients with extensive psoriasis reported becoming depressed, 35% had increased agitation, 30% had difficulty concentrating, and 23% had changes in eating habits as a result of itch [30]. Another questionnaire identified itch severity as a predictor of depressive symptoms, global distress, and sleep impairment in patients with psoriasis [31]. In a study that specifically focused on the link between the psychosocial and dermatologic correlation of itch in inpatients with psoriasis, severity of itch correlated with the degree of depression, and there was also a correlation between changes in depression and itch following treatment [32]. Sleep disturbance has also been found to be more frequent in patients with psoriasis who experienced itch, versus those who did not, with 34% of patients with sleep disturbances reporting that their sleep problems were caused by itch. Sleep impairment and itch were both associated with lower quality of life in patients with psoriasis [33].

Therefore, it is perhaps not surprising that, in the current analysis, improvements in itch associated with tofacitinib treatment had such a significant effect on DLQI in patients with PsA. Interestingly, changes in itch and PGA-PsO appeared to account for the whole treatment effect on DLQI. Given the apparent wide-ranging effects that itch can have on patients, it is possible that relief of itch following tofacitinib treatment may contribute to improved HRQoL in numerous ways. As previously stated, patients with PsA frequently report that itch causes sleep disturbance, and while tofacitinib has been associated with improvements in fatigue in patients with PsA, specific assessments of sleep quality, and its association with itch relief following tofacitinib treatment, have to our knowledge not yet been reported [18,19]. More detailed investigations are required to assess whether improvements in itch in patients with PsA treated with tofacitinib may correlate with changes in patient HRQoL outcomes.

Although the current analysis included data only to Month 3 of treatment, improvements in itch, PGA-PsO, and DLQI associated with tofacitinib were maintained up to Month 12 in OPAL Broaden, and Month 6 in OPAL Beyond [20]. It would be interesting to perform a mediation analysis on data from later time points, to determine whether itch continues to be the main mediator of the effect of tofacitinib on improvements in DLQI.

A similar mediation analysis was previously used to assess the effect of tofacitinib on pain reduction in patients with PsA, specifically with regards to changes in inflammation, with itch shown to be the main mediator of the treatment effect on pain [34]. In patients with rheumatoid arthritis, comparison of the effects of baricitinib, a JAK inhibitor, and adalimumab, a TNFi, both used with concomitant methotrexate, identified a potential difference in the way these treatments alleviate pain [35]. Although baricitinib had a greater overall effect on pain relief than adalimumab in patients with rheumatoid arthritis, changes in inflammation had a larger contribution to the effect of adalimumab [35]. In the context of the current analysis, it may, therefore, also be valuable to compare the effects of treatments other than tofacitinib on dermatology-specific HRQoL in patients with PsA, to assess whether the strong relationship between itch and DLQI is specific to the effect of tofacitinib.

This post-hoc analysis was limited to data obtained within 3 months of treatment initiation, as patients receiving placebo advanced to tofacitinib treatment at Month 3 in the phase III studies. While the mediation models developed here demonstrate how well the data support, or are consistent with, the hypothesized causal relationships among tofacitinib treatment, itch, PGA-PsO, and DLQI, they do not definitively prove that causal relationships exist. Additionally, other explanatory variables not assessed in this study may also mediate the effect of tofacitinib treatment on improved DLQI. Despite this, we are not aware of any potential confounders, measured or unmeasured, that may alter the results and conclusion.

## 5. Conclusions

Dermatology-focused mediation modeling showed that the majority of the overall effect (~80%) of tofacitinib treatment on DLQI was mediated by improvements in itch, with ~20% mediated by improvements in PGA-PsO (represented by erythema, induration, and scaling). Further research is needed to explore how the relief of itch contributes to the observed improvements in dermatology-specific HRQoL.

## Data Availability

Upon request, and subject to certain criteria, conditions, and exceptions (see https://www.pfizer.com/science/clinical-trials/trial-data-and-results for more information), Pfizer will provide access to individual de-identified participant data from Pfizer-sponsored global interventional clinical studies conducted for medicines, vaccines, and medical devices (1) for indications that have been approved in the US and/or EU, or (2) in programs that have been terminated (i.e., development for all indications has been discontinued). Pfizer will also consider requests for the protocol, data dictionary, and statistical analysis plan. Data may be requested from Pfizer trials 24 months after study completion. The de-identified participant data will be made available to researchers whose proposals meet the research criteria and other conditions, and for which an exception does not apply, via a secure portal. To gain access, data requestors must enter into a data access agreement with Pfizer.

## Supplementary Materials

The following are available online at https://www.mdpi.com/article/10.3390/jcm10184081/s1, Figure S1: Effects of tofacitinib treatment on DLQI in the initial mediation model, using data from OPAL Broaden. (a) Indirect effect via itch (measured by ISI); (b) indirect effect via PGA-PsO; (c) direct effect, Figure S2: Effects of tofacitinib treatment on DLQI in the initial mediation model, using data from OPAL Beyond. (a) Indirect effect via itch (measured by ISI); (b) indirect effect via PGA-PsO; (c) direct effect, Figure S3: Effects of tofacitinib treatment on DLQI in the re-specified mediation model, using data from OPAL Broaden. (a) Indirect effect via itch (measured by ISI); (b) indirect effect via PGA-PsO, Figure S4: Effects of tofacitinib treatment on DLQI in the re-specified mediation model, using data from OPAL Beyond. (a) Indirect effect via itch (measured by ISI); (b) indirect effect via PGA-PsO.
