# Supplementary material for: Itch as Major Mediator of Effect of Tofacitinib on Health-Related Quality of Life in Psoriatic Arthritis: A Mediation Analysis
Source: J Clin Med. 2021 Sep 9;10(18):4081. doi: 10.3390/jcm10184081 (PMC8472763; doi:10.3390/jcm10184081)
Supplement: Supplementary file 1 [file jcm-10-04081-s001.zip › jcm-1308552-supplementary.pdf]

Supplementary Materials

Figure S1: Effects of tofacitinib treatment on DLQI in the initial mediation model, using data from OPAL Broaden. (**a**) Indirect effect via itch (measured by ISI); (**b**) Indirect effect via PGA-PsO; (**c**) Direct effect.

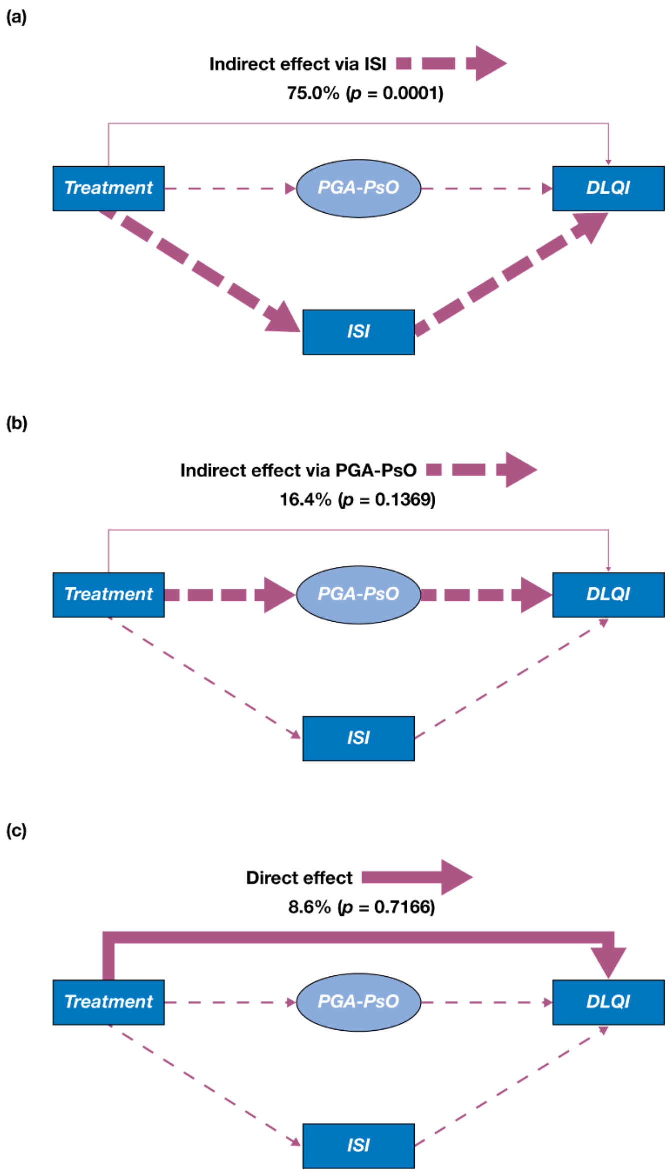

Treatment was represented by a binary variable (tofacitinib 5 mg BID [value of 1] versus placebo [value of 0]); the model included mean scores of each outcome from Months 1 and 3 for every patient with available data from OPAL Broaden; PGA-PsO was a latent variable represented by means of erythema, induration, and scaling. BID, twice daily; DLQI, Dermatology Life Quality Index; ISI, Itch Severity Item; PGA-PsO, Physician Global Assessment of Psoriasis.

Figure S2: Effects of tofacitinib treatment on DLQI in the initial mediation model, using data from OPAL Beyond. (**a**) Indirect effect via itch (measured by ISI); (**b**) Indirect effect via PGA-PsO; (**c**) Direct effect.

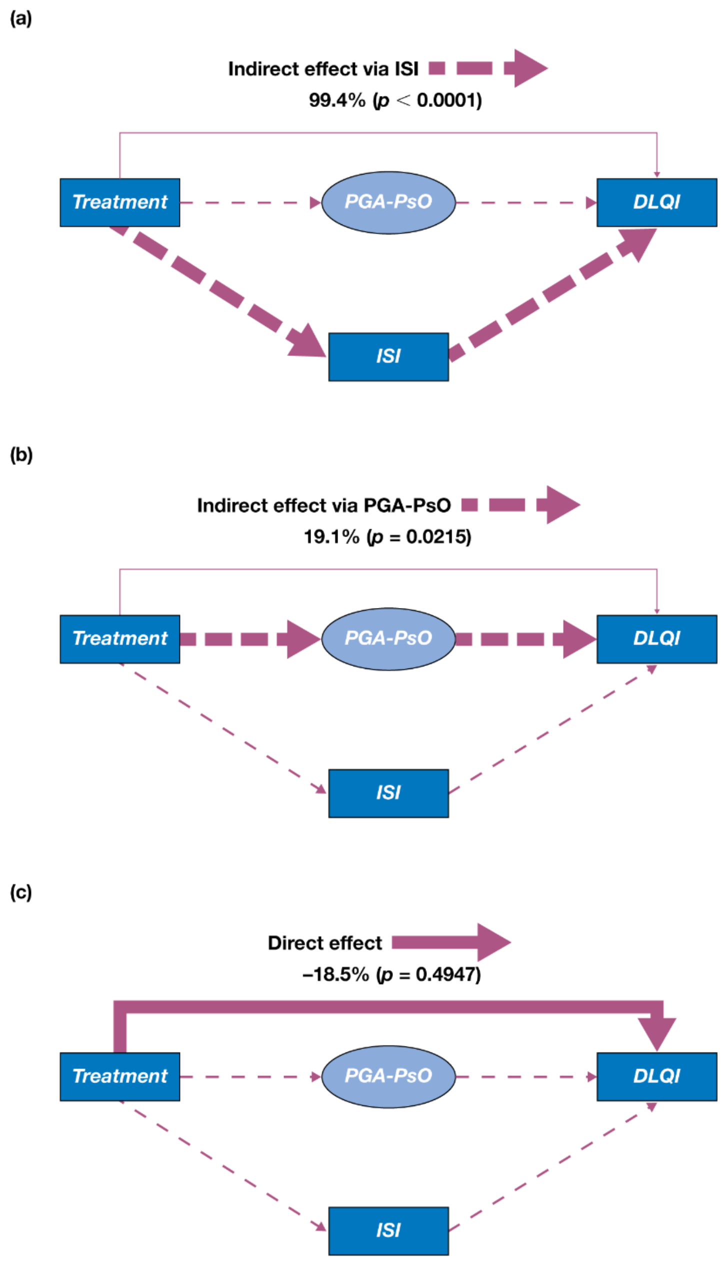

Treatment was represented by a binary variable (tofacitinib 5 mg BID [value of 1] versus placebo [value of 0]); the model included mean scores of each outcome from Months 1 and 3 for every patient with available data from OPAL Beyond; PGA-PsO was a latent variable represented by means of erythema, induration, and scaling. BID, twice daily; DLQI, Dermatology Life Quality Index; ISI, Itch Severity Item; PGA-PsO, Physician Global Assessment of Psoriasis.

Figure S3: Effects of tofacitinib treatment on DLQI in the re-specified mediation model, using data from OPAL Broaden. (**a**) Indirect effect via itch (measured by ISI); (**b**) Indirect effect via PGA-PsO.

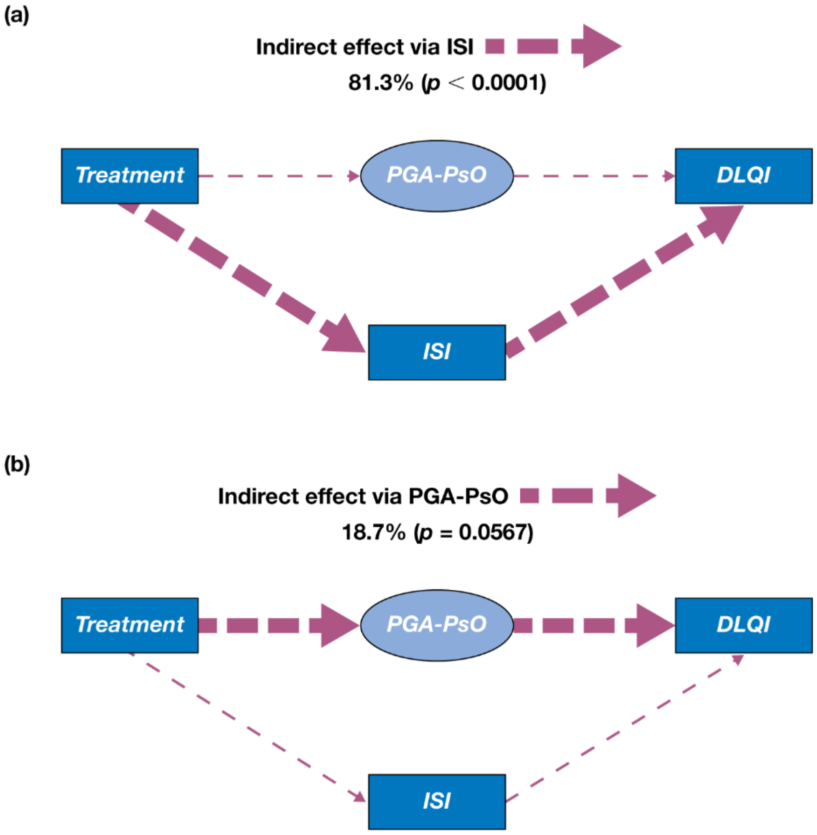

Treatment was represented by a binary variable (tofacitinib 5 mg BID [value of 1] versus placebo [value of 0]); the model included mean scores of each outcome from Months 1 and 3 for every patient with available data from OPAL Broaden; PGA-PsO was a latent variable represented by means of erythema, induration, and scaling. BID, twice daily; DLQI, Dermatology Life Quality Index; ISI, Itch Severity Item; PGA-PsO, Physician Global Assessment of Psoriasis.

Figure S4: Effects of tofacitinib treatment on DLQI in the re-specified mediation model, using data from OPAL Beyond. (**a**) Indirect effect via itch (measured by ISI); (**b**) Indirect effect via PGA-PsO.

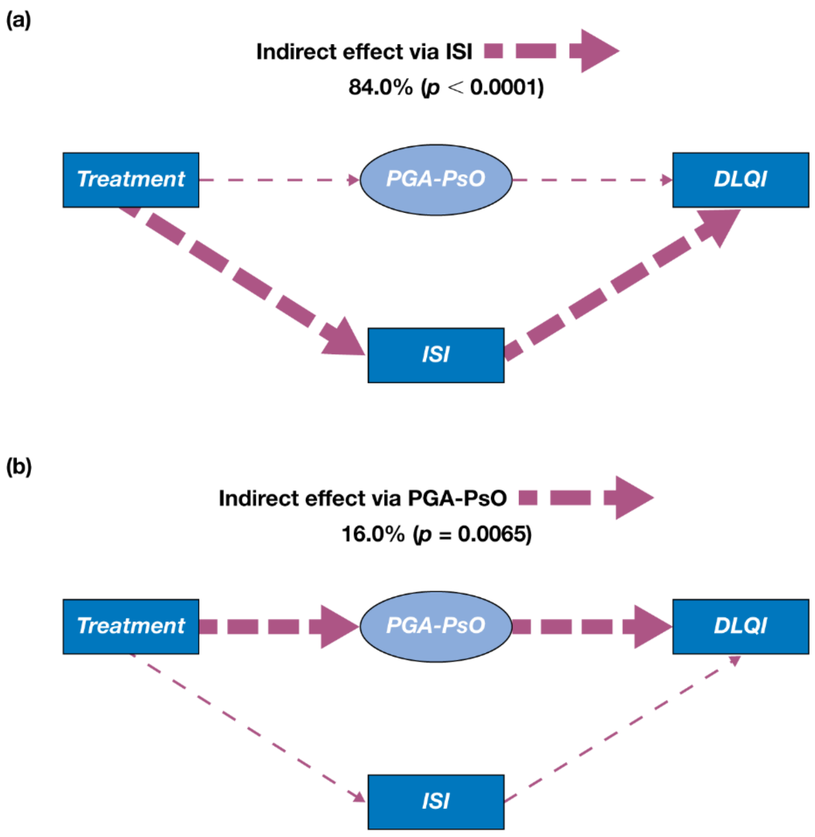

Treatment was represented by a binary variable (tofacitinib 5 mg BID [value of 1] versus placebo [value of 0]); the model included mean scores of each outcome from Months 1 and 3 for every patient with available data from OPAL Beyond; PGA-PsO was a latent variable represented by means of erythema, induration, and scaling. BID, twice daily; DLQI, Dermatology Life Quality Index; ISI, Itch Severity Item; PGA-PsO, Physician Global Assessment of Psoriasis.
